# Effect of seed priming with auxin on ROS detoxification and carbohydrate metabolism and their relationship with germination and early seedling establishment in salt stressed maize

**DOI:** 10.1186/s12870-024-05413-w

**Published:** 2024-07-25

**Authors:** Hasna Ellouzi, Imen Ben Slimene Debez, Souhir Amraoui, Mokded Rabhi, Mohsen Hanana, Nouf M. Alyami, Ahmed Debez, Chedly Abdelly, Walid Zorrig

**Affiliations:** 1 Laboratory of Extremophile Plants (LPE), Centre of Biotechnology of Borj-Cedria (CBBC), P.O. Box 901, Hammam‑Lif, 2050 Tunisia; 2 Laboratory of Bioactive Substances (LSBA), Centre of Biotechnology of Borj-Cedria (CBBC), P. O. Box 901, Hammam‑Lif, 2050 Tunisia; 3 Department of Plant Production, College of Agriculture and Food, Qassim University, Buraydah, Saudi Arabia; 4 Department of Zoology, College of Science, King Saud University, PO Box -2455, Riyadh, 11451 Saudi Arabia

**Keywords:** Auxin, Germination, Seed priming, Signal, Salt stress, Reactive oxygen species

## Abstract

As crucial stages in the plant ontogeny, germination and seedling establishment under adverse conditions greatly determine staple crop growth and productivity. In the context of green technologies aiming to improve crop yield, seed priming is emerging as an effective approach to enhance seed vigor and germination performance under salt stress. In this study, we assess the efficiency of seed priming with indole-3-acetic acid (IAA) in mitigating the adverse effects of salt stress on maize (*Zea mays* L.) seedlings during germination and early seedling stages. In unprimed seeds, salt stress reduced germination indices, and seedling (both radicle and coleoptile) growth, together with decreased tissue hydration. However, seed priming using IAA significantly improved maize salt response, as reflected by the increased seed germination dynamics, early seedling establishment, and water status. Besides, seedlings from IAA-primed seeds showed a higher activity of α-amylase, resulting in increased sugar contents in roots and coleoptiles of salt-stressed plants. Further, IAA-seed priming stimulated the accumulation of endogenous IAA in salt-stressed seedlings, in concomitance with a significant effect on reactive oxygen species detoxification and lipid peroxidation prevention. Indeed, our data revealed increased antioxidant enzyme activities, differentially regulated in roots and coleoptiles, leading to increased activities of the antioxidant enzymes (SOD, CAT and GPX). In summary, data gained from this study further highlight the potential of IAA in modulating early interactions between multiple signaling pathways in the seed, endowing maize seedlings with enhanced potential and sustained tolerance to subsequent salt stress.

## Introduction

Germination is a pivotal phase in the plant life cycle and is integral to crop production. It is a highly dynamic process encompassing the activation of enzymatic systems (like proteases and amylases), energy acquisition, and the synthesis of new hormones and regulators [1]. As such, boosting seed quality (both viability and vigor) is of major significance, not only for the establishment of agricultural crops, but also to cope with environmental challenges [2].

Salinity is a global abiotic stressor that hampers seed germination and restricts plant growth and development worldwide [3]. While salinity affects all plant growth stages, the first stages, specifically germination and seedling establishment, are particularly impacted [4]. Seed germination is impaired by salt ions, predominantly due to the diminished osmotic potential of the external environment, thereby inhibiting seed imbibition and embryonic growth [5]. Besides, high Na^+^ concentrations hinder seed germination by limiting the supply of energy and metabolites [1]. The presence of reactive oxygen species (ROS) during salt-induced stress, resulting in diminished photosynthesis [6]. Seed germination is also sensitive to salt stress-induced ROS damage via lipid peroxidation. Owing to excessive Na^+^ uptake, membrane permeability and seed cell architecture undergo modifications, culminating in oxidative stress in seeds, thereby diminishing seed vigor [7], and ultimately compromising quality and crop yield [8]. Currently, germination and emergence are arguably the most crucial indicators of salt stress resilience. Thus, there is a need for effective and environmentally friendly strategies to enable successful seed germination in saline conditions.

Despite the numerous attempts to enhance salt tolerance using traditional breeding methods, the intricate nature of salt tolerance mechanisms, variation in plant responses to salinity across growth stages, and particularly the absence of ideal selection criteria for salt tolerance led to limited progress [4]. Thus, agriculture requires alternative strategies that are economically viable, environmentally benign, and notably cost-effective. Recently, seed priming has emerged as a potent technology to counteract abiotic stress effects, including salinity, affordable for small farmers and researchers [9]. Seed priming relies on exposing seeds to various physiological agents, achieving controlled hydration without radicle emergence, followed by seed drying [10]. Seed priming can be performed using diverse agents such as water, salts, osmotic components, nutrients, hormones, or biostimulants [11]. The choice of these solutions is influenced by the agent type, crop species, seed quality, and the specific stress in question [6]. While priming initiates-controlled hydration of seeds, fostering early developmental cues, it also induces mild stress within the seed, thereby enhancing plant resilience to subsequent adversities [12]. Pre-exposing seeds to mild stress leaves an indelible mark, activating a myriad of signaling networks in primed seeds. Such preliminary occurrences are swiftly recalled, bolstering plant reactions to impending stresses [11, 13].

Apart from their role in regulating plant growth and development, especially under environmental constraints, phytohormones, including cytokinins (CK), gibberellins (GA), brassinosteroids (BRs), ethylene (ET), jasmonic acid (JA), auxin (indole-3-acetic acid, IAA), salicylic acid (SA), abscisic acid (ABA), and strigolactone (SL), are potentially considered as potent seed priming agents [14]. Several studies have highlighted GA role in activating physiological and biochemical mechanisms that contribute to salt stress tolerance [15, 16]. Moreover, phytohormones do not operate separately; they function within a complex signaling network combined with other plant molecular signaling pathways [17]. For instance, treatment of *Avena sativa* seeds with GA_3_ mitigated salt stress toxicity by stimulating germination, water uptake, and seedling growth [18]. In a previous study, we also showed such a beneficial effect of GA_3_ seed treatment in cauliflower plants under severe salinity [7]. Similarly, priming wheat seeds with GA_3_ significantly reduced Na^+^ uptake and simultaneously stimulated the activities of key enzymes primarily involved in amino acid biosynthesis [19]. GA_3_ has also been reported to increase ABA-catabolizing enzyme activities, leading to reduced ABA levels, thus promoting seed germination [17]. Brassinosteroids (BRs) have also been shown to enhance defense mechanisms against stresses [20]. It is also worth mentioning that (SA), a prominent signal transducer and messenger, can markedly influence plant stress responses, when used as a seed priming agent [14]. Intriguingly, pretreating *Vicia faba* seeds improved overall germination attributes under salt stress [21]. Recently, we emphasized that SA, when present within barley seeds, orchestrates interactions among the fundamental elements of salt stress signaling pathways, such as ion homeostasis, redox equilibrium, osmoregulators, and ROS scavenger systems, collectively contributing to salinity tolerance in barley seedlings [22]. We also observed that combined seed priming with ethylene and H_2_O_2_ considerably diminished oxidative stress damage in *Arabidopsis thaliana* seedlings under intense salinity [22].

Indole-3-acetic acid (IAA), the most extensively researched natural auxin crucial for cell division and elongation, has also been effectively used in seed priming techniques to enhance plant tolerance to various stresses [23]. Endogenous enrichment of seeds with auxin (IAA) via priming was suggested to promote salt tolerance and ultimately grain yield [24]. In this regard, priming *Triticum aestivum* grains with IAA mitigated the detrimental effects of salinity by restoring germination, hormonal balance, and CO_2_ assimilation, leading to enhanced yields [24]. Pre-sowing wheat seeds with IAA was also found to stimulate growth and photosynthetic activities under saline conditions [25]. Additionally, priming seeds with IAA improved salt tolerance in winter wheat when grown under salinity, notably by modulating ion transport from roots to shoots [26]. Interactions between IAA, JA, and ABA were observed in white clover species subjected to salt stress [27]. In this regard, Fahad et al. [28] reported that auxin-primed seeds increased ABA activities to promote plant tolerance to salt stress. In another study, auxins promoted root elongation under saline conditions, when interacting with ET [29].

Aside from phytohormones, auxin engages in cross-talk with various other components during seed germination, contributing to the intricate regulation of this crucial developmental process. One notable interaction occurs between auxin and carbohydrates such as sugars. Auxin signaling can influence sugar metabolism by regulating the expression of genes involved in sugar biosynthesis, transport, and metabolism. Conversely, sugars can modulate auxin transport and signaling, potentially impacting root growth, nutrient uptake, and stress responses [30]. Furthermore, it has been reported that auxin can interact with sugars under salt stress by regulating the expression and activity of α-amylase, key enzyme involved in carbohydrate metabolism [31]. Thus, by modulating α-amylase activity, auxin can influence starch degradation, leading to changes in sugar metabolism and allocation.

Besides phytohormones and carbohydrates, IAA engages in cross-talk with various other components during seed germination, contributing to the regulation of this crucial developmental process. One notable interaction occurs between auxin and reactive oxygen species (ROS) [32]. For instance, Devireddy et al. [33] provided a comprehensive review highlighting the pivotal role of ROS as effective regulators of phytohormone signaling networks. Fu et al. [34] reported that in salt stressed *Arabidopsis thaliana* seedlings, the crosstalk of IAA and ROS is achieved by enhancing the formation of primary root growth through *IAA-Conjugate-Resistant 4* (*IAR4*), which is greatly associated with ROS-mediated auxin distribution. Previously, Iglesias et al. [35] also reported that auxin and ROS dynamically interact for orchestrating the redox balance in Arabidopsis to defend against oxidative stress. Regarding priming, Queiroz et al. [36] found that seed treatment with IAA led to improved activities of the antioxidant enzymes in soybean seedlings when grown under salinity. Recently, Sun et al. [37] showed that soaking of rice seeds with IAA increased the expression levels of antioxidant defense-related genes to maintain a suitable ROS homeostasis during germination process. Hence, ROS function as signal messenger while not triggering oxidative injuries, to enhance the vigor of aged rice seeds.

Each of these examples highlights the potential of hormopriming in enhancing salt tolerance across various plant species. However, given the intricate nature of phytohormone networks in salinity responses, the selection of an apt hormone for the priming process requires deep understanding of the key components interacting with hormones and their roles in amplifying tolerance. Hence, studying phytohormones as seed priming agents during germination and seedling emergence can provide insights into the early responses elicited by priming and salt stress, laying the groundwork for understanding long-term plant resilience to salinity. Given the economic importance of *Zea mays* L., we address here the impact of IAA priming on seeds germinated in saline environments, focusing on germination dynamics and early seedling growth. Special attention was also given to carbohydrate mobilization, oxidative stress biomarkers and antioxidant enzyme activities to elucidate any potential crosstalk between auxin, carbohydrates and ROS homeostasis under salt stress conditions.

## Materials and methods

### Seed priming and salt stress treatment

Maize (*Zea mays* L.; a local variety provided by Baddar seeds, Tunisia) seeds of uniform size were disinfected using a diluted sodium hypochlorite (NaOCl) solution for 5 min and subsequently rinsed three times with distilled water. The seeds were then immersed in a 50 ppm solution of indole-3-acetic acid (IAA) and kept for 8 h in the dark at a temperature of 25°C. The choice of IAA concentration is based on studies [38, 39] demonstrating its efficacy in enhancing seed germination and seedling vigor, without causing phytotoxic effects. Seeds that weren’t subjected to the priming process served as the control group. Post priming, both the unprimed seeds (UPS) and the IAA-primed seeds (PS IAA) were placed to germinate on moistened double-layered Petri dishes. The dishes were positioned randomly in a dark environment maintained at 22°C. Each Petri dish (20 seeds per Petri dish) was imbibed on alternate days with 5 ml distilled water containing or not 100 mM NaCl. Every treatment was replicated biologically three times. On day 9 of germination, seedlings (at the coleoptile stage) were sampled and used for growth, physiological and biochemical analyses.

### Germination indices

Seed germination traits were assessed when germinated root tip broke and the visible radicle appeared. The germination indices of primed and unprimed seeds of *Z. mays* L. sown in saline and non-saline media considered the germination percentage (G %), germination index (GI), coefficient of velocity of germination (CVG) and the germination rate index (GRI), by using the method of Kader [40] as following:

$$\mathrm G\%=\left(\mathrm{Number}\;\mathrm{of}\;\mathrm{germinated}\;\mathrm{seeds}/\mathrm{Total}\;\mathrm{number}\;\mathrm{of}\;\mathrm{seeds}\right)\times100.$$

$$\mathrm{GI}=\sum\left(\mathrm{Ti}\times\mathrm{Ni}\right)$$

$$\mathrm{CVG}=\frac{\sum\mathrm{Ni}\times100}{\sum\mathrm{Ti}\;\mathrm{Ni}}$$

where Ti and Ni are respectively the number of days after seed sowing and the number of seeds germinated on day i. GRI = G1/1 + G2/2 + ⋯ + Gx/x.

Where G1 indicates the percentage of germination on day 1, G2 is the germination percentage on day 2, and Gx indicates the germination percentage on the final day of counting.

### Seedling parameters

Root and coleoptile length (cm plant^−1^) were daily measured until 9 d of germination (9 replicates per treatment). At the end of the experiment, seedlings were collected, washed in distilled water, dried on filter paper, and separated in coleoptiles (shoots) and roots. After their respective fresh weight (FW) were determined, samples were dried at 60°C until reaching constant weights and dry weights (DW) were recorded. To assess seedlings water status, hydration (H) of roots and coleoptiles was determined as follows:

$$\mathrm H\;\left(\mathrm{ml}\;{\mathrm H}_2\mathrm O/\mathrm g\;\mathrm{DW}\right)=100\times\left(\mathrm{fresh}\;\mathrm{weight}-\mathrm{dry}\;\mathrm{weight}\right)/\mathrm{dry}\;\mathrm{weight}$$

### Total soluble sugars and α-amylase activity determination

Total soluble sugars in roots and coleoptiles of maize seedlings were determined according to the method described by Shields et al. [41]. Dried root and coleoptile samples (25 mg) were ground and twice extracted to isolate soluble sugars in hot 80% ethanol. Then, samples were centrifuged (3000 rpm for 20 min). Soluble sugar content was analyzed using a spectrophotometer at 640 nm using the enthrone assay standardized to glucose and expressed on a dry weight basis (mg g^−1^ DW).

Concerning α-amylase activity (EC 3.2.1.2), fresh seedling samples including roots and coleoptiles were ground with liquid nitrogen and homogenized in a mortar with 0.1 mol L^−1^ TRIS–HCL buffer (pH 7.2) containing 0.1 mol L^−1^ NaCl and 10 mmol L^−1^ CaCl_2_ and centrifuged for 10 min at a temperature of 4 ºC. The extract was incubated in a water bath for 15 min at 70 ºC. Enzyme quantification was achieved using enzyme extract, sodium acetate buffer (100 mM), calcium chloride (5 mM, pH 5.0) and starch solution. Samples remained in a water bath at 40°C during 15 min. The reaction was stopped by adding the 3,5-dinitrosalicyclic acid (DNS) solution and the samples were kept in a water bath at 95°C for 5 min. The absorbance was performed in a spectrophotometer at 540 nm and results were expressed in units per milligram (U mg^−1^) proteins.

### Estimation of indole acetic acid content

Root and coleoptile IAA content was assessed using Salkowski’s procedure and commercial IAA standard [42]. The supernatant was combined with Salkowski’s reagent and left for 30 min at 25°C. Subsequently, the absorbance was read at 530 nm and the IAA content was expressed as ng g^−1^ FW.

### Determination of hydrogen peroxide and superoxide anion production

H_2_O_2_ content in roots and coleoptiles was assessed using the method of Junglee et al. [43]. The absorbance was read at 390 nm, and H_2_O_2_ content was determined by preparing a calibration curve with H_2_O_2_ standard solutions and expressed as µmol/g FW. Superoxide content of root and coleoptile samples from maize was determined according to Kumar et al. [44] and the absorbance was recorded at 540 nm. Superoxide radical amount was calculated using the extinction coefficient of 12.8 mM^−1^ cm^−1^ and expressed as μmol g^−1^ FW.

### Determination of protein carbonyl content and lipid peroxidation level

Lipid peroxidation was evaluated by measuring the malondialdehyde (MDA) content (nmol g^−1^ FW) in coleoptiles and roots, as described by Hodge et al. [45]. Protein carbonyl content was estimated by dinitrophenylhydrazine (DNPH) procedure [46]. Root and coleoptile samples were suspended in SDS (6% w/v) and stored for 25 min at room temperature. Subsequently, 10 mM DNPH in trichloroacetic acid (TCA, 1.3 M) was added and the reaction mixture incubated at room temperature (25 °C) for 40 min. Then, the samples were centrifuged at 10.000 *g* for 20 min. Finally, the absorbance was read at 360 nm and cabonylated protein content calculated using the molar extinction coefficient of DNP (17, 530 M^−1^ cm^−1^).

### Protein determination and antioxidant enzyme activities

Proteins and antioxidant enzymes were extracted from frozen coleoptiles and roots using polyvinylpyrrolidone in a phosphate buffer (pH 7.8) containing 10 mM ethylenediamine tetraacetic acid (EDTA), 1 mM dithiothreitol, and 0.1 mM phenyl-methyl-sulfonyl fluoride (PMSF). After centrifugation at 12,000 *g* for 30 min, supernatants were collected and used for protein and enzyme analysis.

Total protein content was evaluated using the Bradford [47] method, with bovine serum albumin as the standard.

Superoxide dismutase (SOD, EC: 1.15.1.1) activity was determined using the method of Scebba et al. [48]. Total catalase (CAT, EC: 1.11.1.6) activity was assayed by measuring the decline in absorbance at 240 nm following Lück’s method [49]. According to the assay of Fielding and Hall [50], guaiacol peroxidase (GPX, EC: 1.11.1.7) activity was analyzed by the increase in absorbance at 470 nm.

### Statistical analysis

The SPSS version 21.0 (SPSS Inc., Chicago, USA) software was used to assess differences between salt treatments for a given priming treatment by analysis of variance (ANOVA) according to Duncan’s multiple range tests (*p* < 0.05). A Principal Component Analysis (PCA) and correlation analysis were performed using the XLSTAT software v. 2014 (Addinsoft, Paris, France), with variables centered on their means and normalized with a standard deviation of 1.

## Results

### Effect of IAA priming on seed germination dynamics

**Fig. 1:**
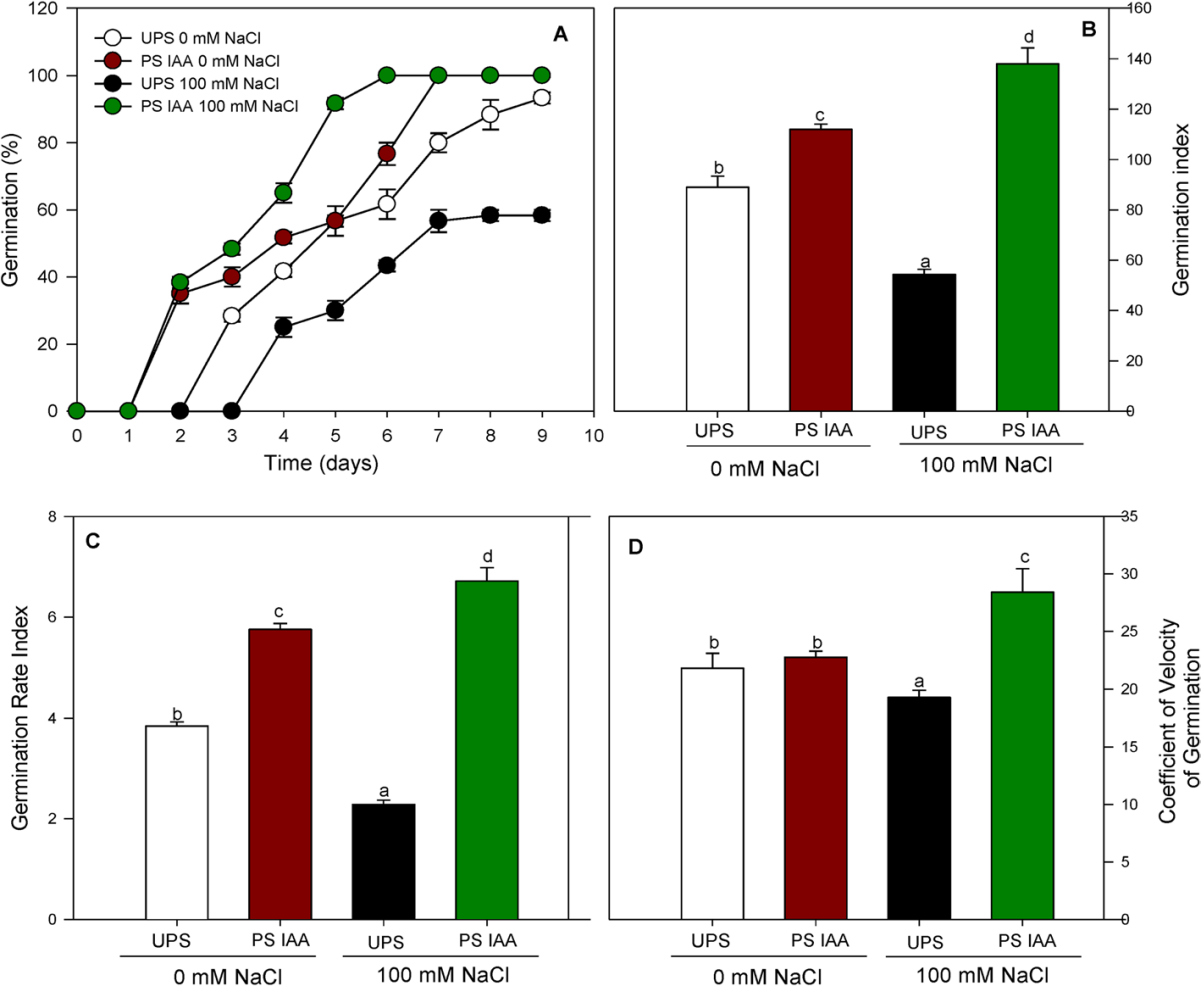
Effect of seed priming with IAA (PS IAA) on the germination percentage (**A**), germination index (**B**), germination rate index (**C**), and coefficient of velocity of germination (**D**) of maize seeds sown under saline (100 mM NaCl) and non-saline (0 mM NaCl) conditions. UPS: UnPrimed Seeds; PS IAA: Primed Seeds with IAA. Means of three replicates ± SE. In each panel, bars marked with the same lowercase letters are not significantly different at *p* ≤ 0.05 (Duncan’s test)

Priming clearly induced faster germination since the first germinated primed seeds appeared on d 2 after sowing in non-saline (0 mM NaCl) or saline (100 mM NaCl) conditions (Fig. 1A). Under salt-free conditions, unprimed seeds (UPS) started to germinate after 3 d of sowing, reaching 93% germination on d 9. In UPS 100 mM NaCl salinity delayed germination, which started on d 4, and reached only 58% germinating on d 9 following sowing. On the contrary, seeds primed with IAA and exposed to 100 mM NaCl displayed the highest germination percentage (100%) by d 6 of sowing (Fig. 1A).

Germination index (GI), germination rate index (GRI), and coefficient of velocity of germination (CVG) were significantly (*p* < 0.05) improved with IAA priming under saline and non-saline conditions. Interestingly, values of these parameters were higher even when germination occurred in saline media, showing 156, 194 and 48% increases for GI (Fig. 1B), GRI (Fig. 1C), and CVG (Fig. 1D) respectively, compared to salt-stressed UPS. It is worth mentioning that salt stress application significantly (*p* < 0.05) reduced the overall germination indices inUPS.

### Effect of IAA priming on the early seedling growth components

#### Root and coleoptile length

**Fig. 2:**
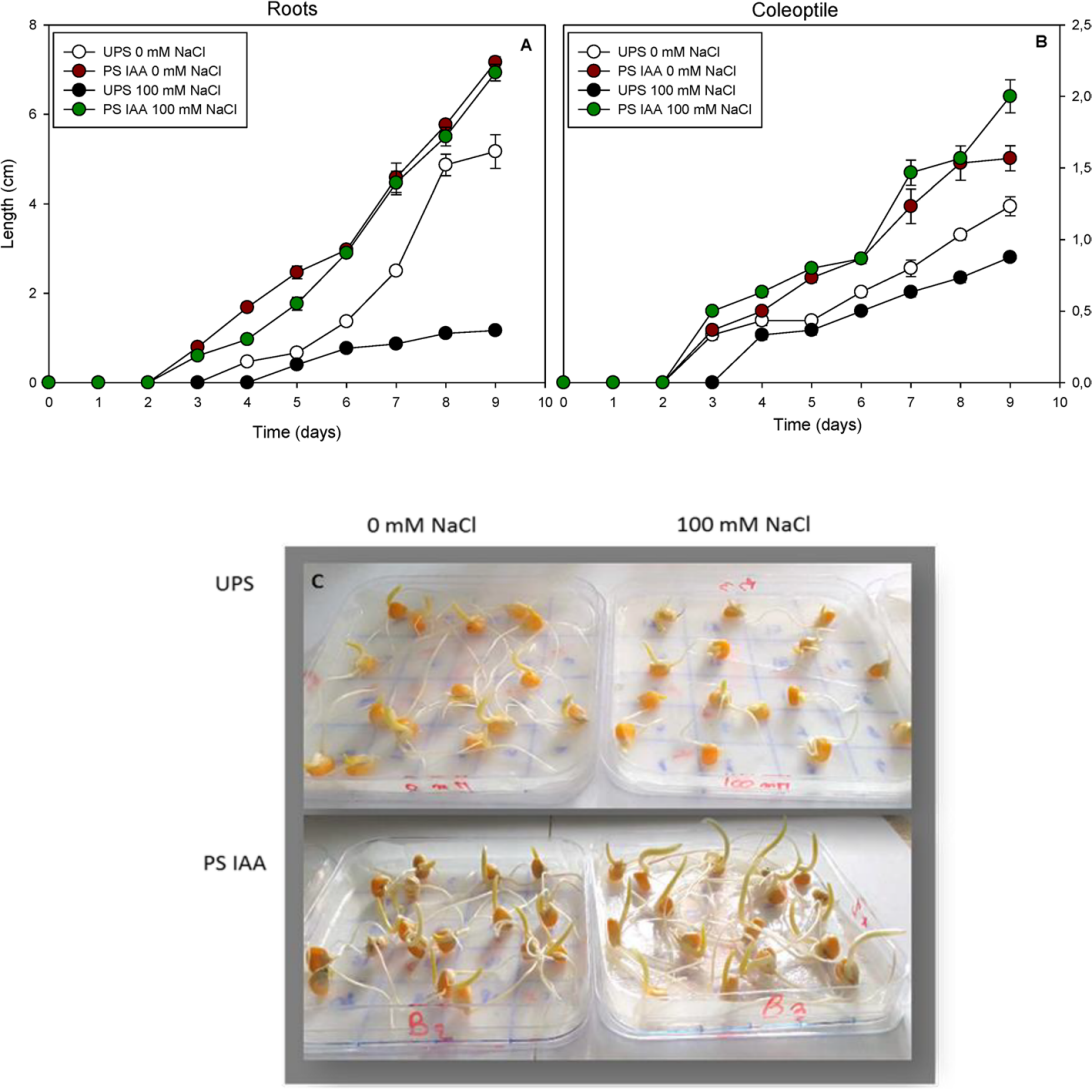
Effect of seed priming on the root (**A**) and coleoptile (**B**) length of maize seeds sown under saline (100 mM NaCl) and non-saline (0 mM NaCl) conditions. Morphological aspect of maize seedlings grown under saline and non-saline conditions for 9 d (**C**). UPS: UnPrimed Seeds; PS IAA: Primed Seeds with IAA. Means of three replicates ± SE

In UPS, salt stress significantly reduced root elongation from the first days of measurement, reaching a 116% decrease as compared to the salt-free conditions at d 9 (Fig. 2A). This was accompanied by a marked decrease (-30%) in coleoptile length (Fig. 2B). Seedlings that germinated from seeds primed with IAA exhibited significantly longer root and coleoptile lengths under both saline and non-saline conditions. Interestingly, salt application significantly stimulated root elongation (by sixfold) (Fig. 2A) and coleoptile length (by threefold) (Fig. 2B) compared to those of UPS germinated in saline media. Besides, the morphological aspect of maize seedlings confirmed the beneficial effect of IAA seed priming, with no records of salt symptoms (Fig. 2C).

#### Root and coleoptile biomass and hydration

**Fig. 3:**
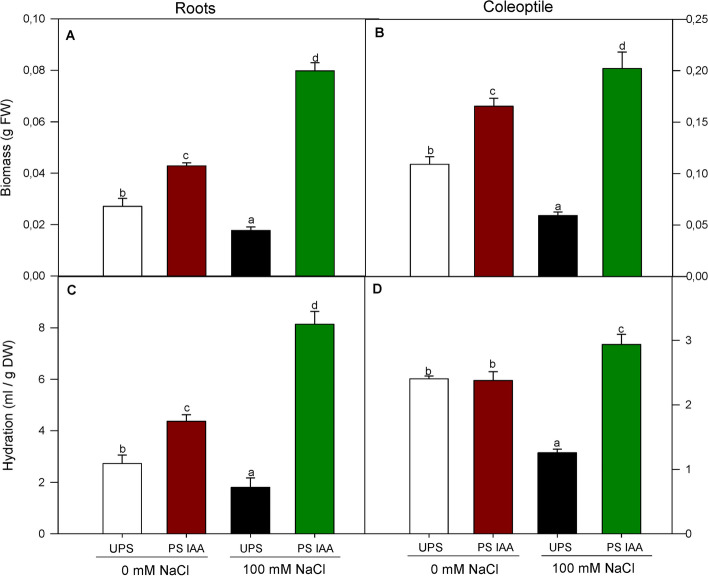
Effect of seed priming on root (**A**) and coleoptile (**B**) biomass and root (**C**) and coleoptile (**D**) hydration of maize seedlings grown under saline (100 mM NaCl) and non-saline (0 mM NaCl) conditions. UPS: UnPrimed Seeds; PS IAA: Primed Seeds with IAA. Maize seedlings grown under saline and non-saline conditions for 9 d. Means of three replicates ± SE. In each panel, bars marked with the same lowercase letters are not significantly different at *p* ≤ 0.05 (Duncan’s test)

Under UPS conditions, salt treatment significantly decreased root and coleoptile biomass by 63 and 60%, respectively, compared to 0 mM NaCl (Fig. 3A, B). When seeds were IAA-primed without NaCl treatment (PS IAA, 0 mM NaCl), both root and coleoptile growth (Fig. 3A, B) improved significantly by 55 and 65%, respectively, as compared to UPS at 0 mM NaCl. However, seedlings from PS IAA germinating under 100 mM NaCl exhibited the highest root (+ 90%) (Fig. 3A) and coleoptile (+ 25%) (Fig. 3B) biomass, compared to 0 mM NaCl condition.

Seedlings from UPS which germinated in saline media displayed the lowest hydration levels in both roots (-34%) and coleoptiles (-52%) compared to UPS at 0 mM NaCl (Fig. 3C, D). Again, priming maize seeds with IAA improved water uptake during germination, irrespective of salt treatment. In the case of salt stress, root (the first organ in direct contact with salinity) hydration increased by 84% compared to roots grown under non-saline media (Fig. 3C). Similar trends were observed in coleoptile hydration, with an increase of 27%, compared to those of PS IAA germinated under 0 mM NaCl (Fig. 3D).

### Effect of IAA priming on the early total sugar content and α-amylase activity

**Fig. 4:**
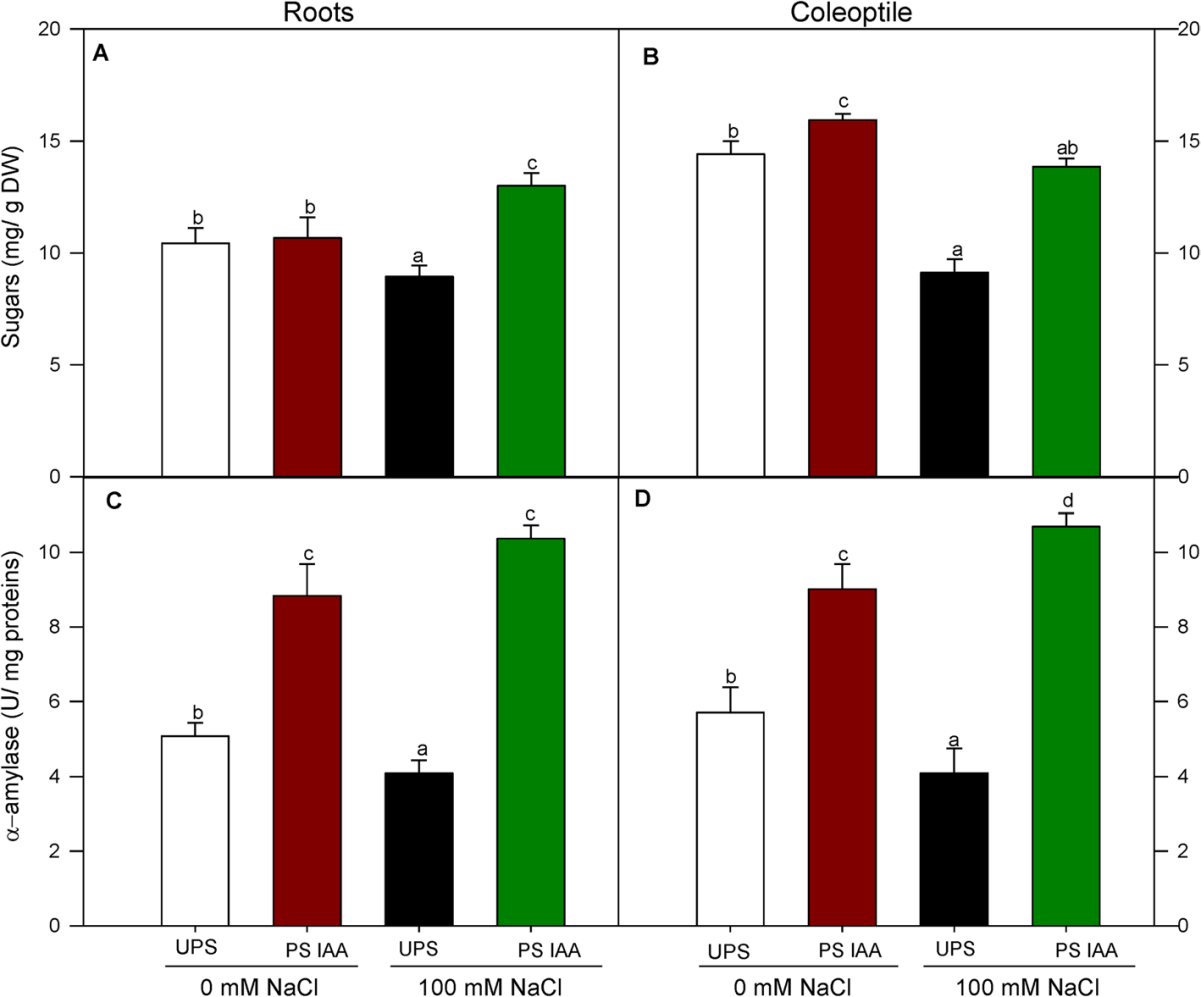
Effect of seed priming on root (**A**) and coleoptile (**B**) sugars content and root (**C**) and coleoptile (**D**) α-amylase activity of maize seedlings grown under saline (100 mM NaCl) and non-saline (0 mM NaCl) conditions. UPS: UnPrimed Seeds; PS IAA: Primed Seeds with IAA. Maize seedlings grown under saline and non-saline conditions for 9 d. Means of three replicates ± SE. In each panel, bars marked with the same lowercase letters are not significantly different at *p* ≤ 0.05 (Duncan’s test)

Sugar content of both roots (Fig. 4A) and coleoptiles (Fig. 4B) were significantly lower in the presence of NaCl, about 1.2-and 1.6-fold respectively. In seeds primed with IAA, root sugar content increased maximally (+ 45%) when germination took place in saline media as compared to unprimed state combined with salinity (Fig. 4A). Coleoptiles from IAA-primed seeds also exhibited a 55.5% of increase in sugar content, relatively to unprimed salt stressed seedlings (Fig. 4B).

Salt stress caused a marked decrease in α-amylase activity in both maize roots (by 1.25 times) and coleoptiles (1.5 times) as compared to their counterparts grown under non-saline conditions (Fig. 4C, D). Similarly, IAA seed priming significantly increased (*p* < 0.05) α-amylase activity in both roots and coleoptiles under salinity. In this context, the level of α-amylase activity was highest in roots (+ 160%) from PS IAA grown under salt stress as compared to their homologues from UPS (Fig. 4C). This was also true for coleoptile α-amylase, which also exhibited the highest activity (+ 175%), relatively to those of UPS treatment (Fig. 4D).

### Root and coleoptile auxin content

**Fig. 5:**
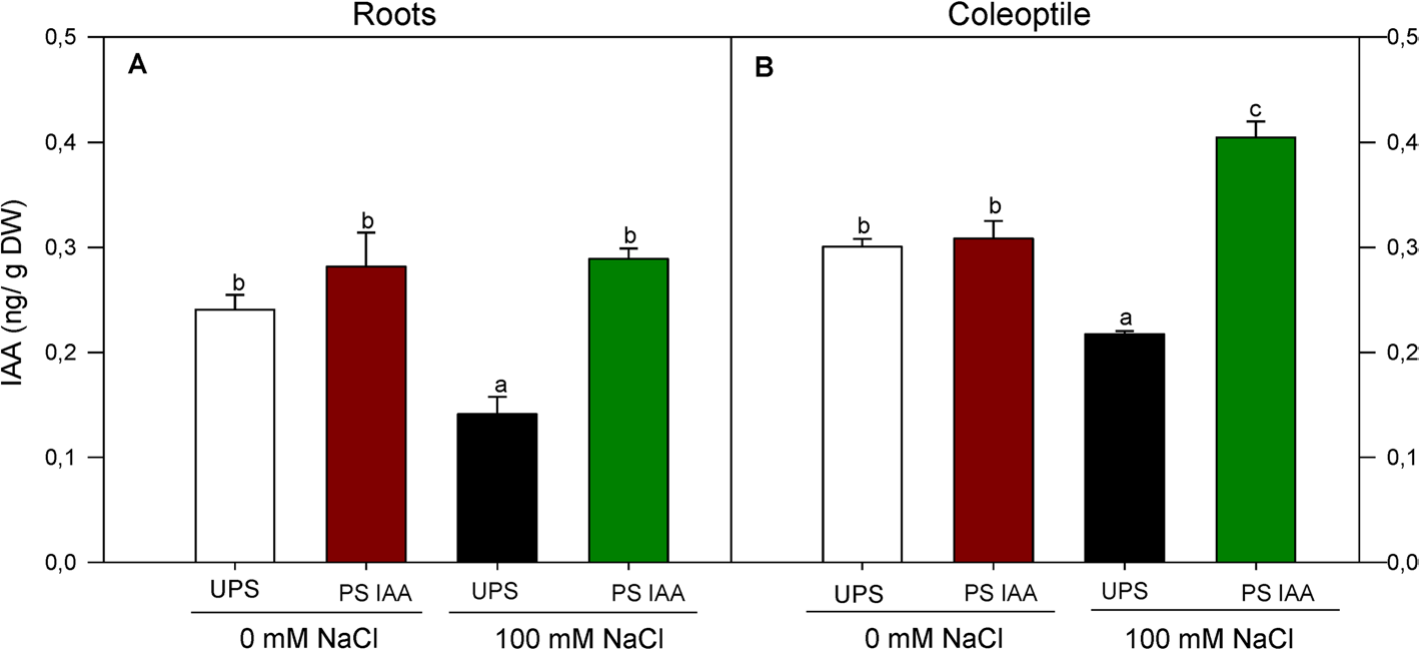
Effect of seed priming on root (**A**) and coleoptile (**B**) auxin content of maize seedlings grown under saline (100 mM NaCl) and non-saline (0 mM NaCl) conditions. UPS: UnPrimed Seeds; PS IAA: Primed Seeds with IAA. Maize seedlings were grown under saline and non-saline conditions for 9 d. Means of three replicates ± SE. In each panel, bars marked with the same lowercase letters are not significantly different at *p* ≤ 0.05 (Duncan’s test)

There was a strong and significant (*p* < 0.05) decrease in IAA content in both roots and coleoptiles, from UPS germinated under saline conditions, being reduced 42% in roots (Fig. 5A) and 30% in coleoptiles (Fig. 5B), as compared to UPS grown under non-saline conditions. By contrast, in IAA primed seeds germinating in saline media, roots showed a 107% increase in IAA content (Fig. 5A), followed by the coleoptiles (+ 82%) (Fig. 5B).

### Effect of IAA priming on early H_2_O_2_ and superoxide accumulation

**Fig. 6:**
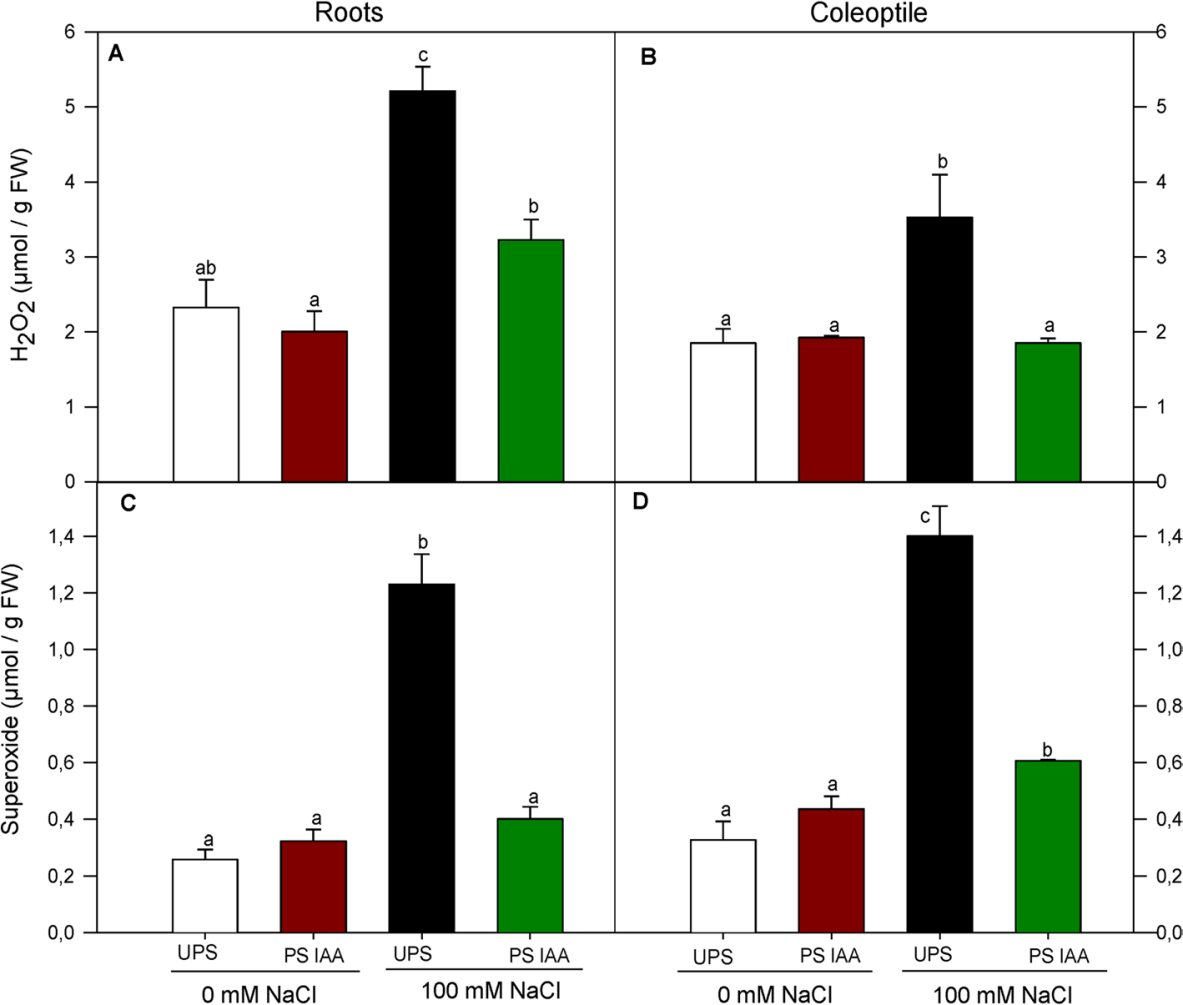
Effect of seed priming on H_2_O_2_ content in roots (**A**) and coleoptiles (**B**) and superoxide content in roots (**C**) and coleoptiles (**D**) of maize seedlings grown under saline (100 mM NaCl) and non-saline (0 mM NaCl) conditions. UPS: UnPrimed Seeds; PS IAA: Primed Seeds with IAA. Maize seedlings were grown under saline and non-saline conditions for 9 d. Means of three replicates ± SE. In each panel, bars marked with the same lowercase letters are not significantly different at *p* ≤ 0.05 (Duncan’s test)

Hydrogen peroxide (H_2_O_2_) and superoxide anion (O_2_^.−^) are reliable indicators of oxidative stress in both primed and unprimed seeds in response to salt stress. There was a considerably higher H_2_O_2_ accumulation in roots (by + 124%) (Fig. 6A) and coleoptiles (by + 90%) (Fig. 6B) from UPS grown under salt treatment. In contrast, H_2_O_2_ content was significantly (*p* < 0.05) reduced by 38% in roots and by 48% in coleoptiles from IAA-primed seeds under saline conditions as compared with UPS grown under salt stress (Fig. 6A, B).

Superoxide content showed a similar trend to H_2_O_2_. Generally, UPS-grown seedlings exhibited higher superoxide levels under salt stress conditions in both roots (by + 370%) and coleoptiles (by 325%) (Fig. 6C, D) as compared to UPS germinating at 0 mM NaCl. Despite germinating under 100 mM NaCl, seedlings from IAA primed seeds showed lower superoxide accumulation. This was especially true for roots (by 67.5%) for which value was statistically similar to that of the control (Fig. 6C). In salt stressed coleoptiles from IAA primed seeds, superoxide accumulation declined by 57% compared to those from UPS and grown in saline media (Fig. 6D).

### Effect of IAA priming on lipid peroxidation and protein carbonyl content

**Fig. 7:**
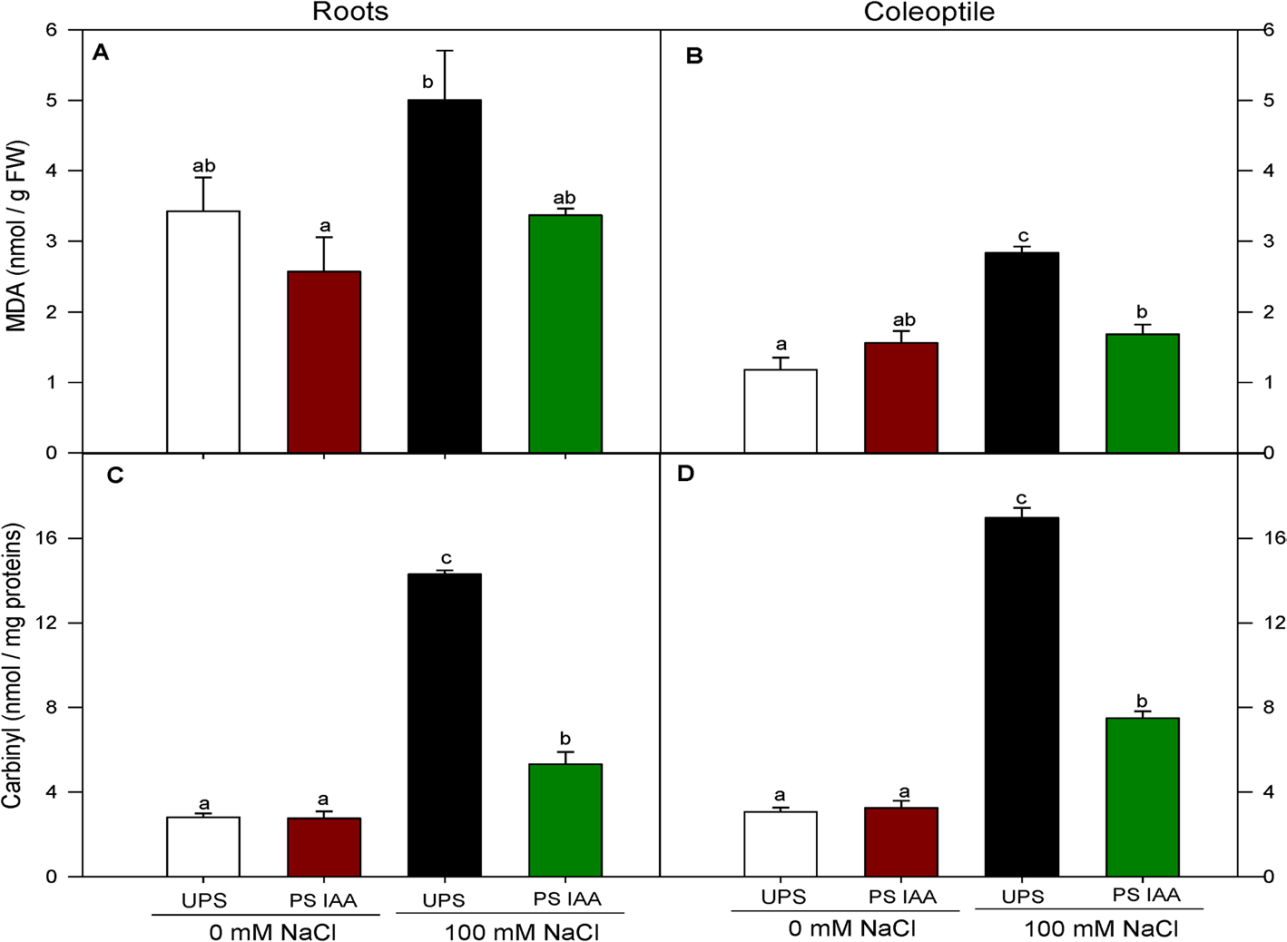
Effect of seed priming on MDA content in roots (**A**) and coleoptiles (**B**) and carbonyl content in roots (**C**) and coleoptiles (**D**) of maize seedlings grown under saline (100 mM NaCl) and non-saline conditions (0 mM NaCl). UPS (UnPrimed Seeds), PS IAA (Primed Seeds with IAA). Maize seedlings were grown under saline and non-saline conditions for 9 d. Means of three replicates ± SE. In each panel, bars marked with the same lowercase letters are not significantly different at *p* ≤ 0.05 (Duncan’s test)

To further explore the extent of membrane lipid peroxidation and protein oxidation damages caused by ROS accumulation, MDA production and carbonyl content were assessed respectively, in combined priming and salinity conditions. Under salt conditions (Fig. 7A, B), MDA content markedly increased in both roots (by 46%) and coleoptiles (by 142%) from UPS. Again, priming seeds with IAA significantly (*p* < 0.05) reduced MDA accumulation, even under salt treatment, in both roots (by 33%) (Fig. 7A) and coleoptiles (by 41%) (Fig. 7B), compared to IAA primed seeds germinating at 0 mM NaCl.

Salinity had a pronounced effect on protein oxidation as estimated by carbonyl formation, in maize seedlings from UPS. This parameter was significantly increased in both roots (by 400%) and coleoptiles (by 467%) from UPS seeds germinating under salt stress conditions (Fig. 7C, D), as compared to non-stressed UPS seedlings. Similar to lipid peroxidation, protein oxidation was markedly reduced following seed priming with IAA. For example, roots from seeds treated with IAA and germinated in saline media exhibited a 63% decrease in carbonyl content (Fig. 7C), while a 56% of decrease was recorded in coleoptiles from IAA primed seeds (Fig. 7D), compared to seedlings from salt stressed UPS.

### Effect of IAA priming on the early protein content and antioxidant defense systems

**Fig. 8:**
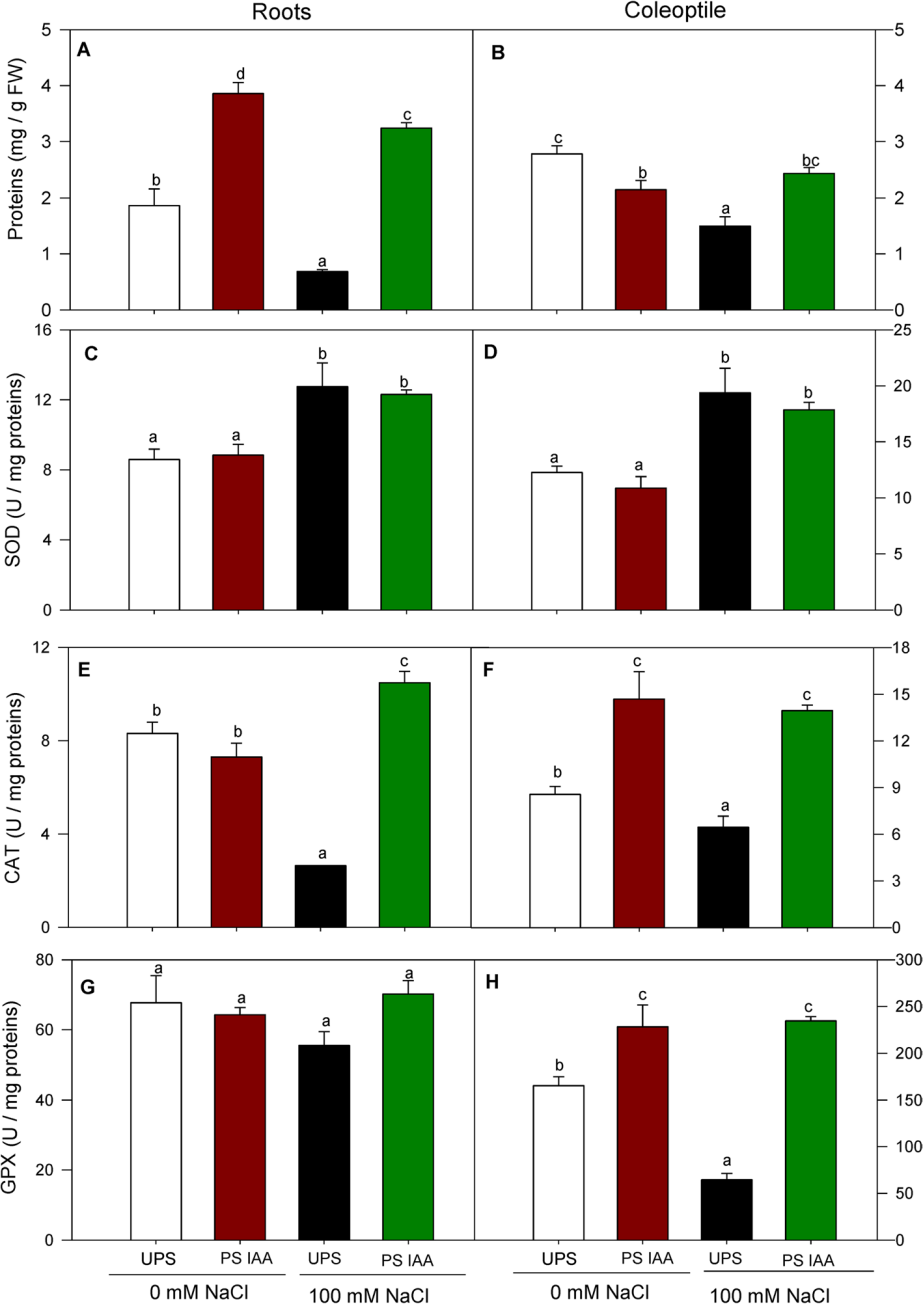
Effect of seed priming on protein content and SOD, CAT, and GPX activities in the roots (**A**, **C**, **E** and **G**) and coleoptiles (**B**, **D**, **F** and **H**) of maize seedlings grown under saline (100 mM NaCl) and non-saline conditions (0 mM NaCl). UPS (UnPrimed Seeds); PS IAA (Primed Seeds with IAA). Maize seedlings were grown under saline and non-saline conditions for 9 d. Means of three replicates ± SE. In each panel, bars marked with the same lowercase letters are not significantly different at *p* ≤ 0.05 (Duncan’s test)

Under salt stress, protein content significantly decreased in roots (by 63%) and coleoptiles (by 47%) arising from UPS (Fig. 8A, B). However, pretreating seeds with IAA strongly offest salt stress effect on protein accumulation. Roots and coleoptiles from IAA-primed seeds exhibited a 377% and 64% increase in protein content, respectively (Fig. 8A, B), compared to the unprimed treatment combined with NaCl.

Regarding the antioxidant defense, when seeds were primed with IAA without salt addition, SOD activity did not show significant (*p* > 0.05) variation in both roots and coleoptiles compared to the unprimed regime (Fig. 8C, D). Under salt treatment, roots and coleoptile SOD activities from both PS IAA and UPS significantly (*p* < 0.05) increased (Fig. 8C, D). Opposite trends were observed in CAT activity profile; seedlings from unprimed seeds and germinated in saline media exhibited significantly (*p* < 0.05) lower CAT activities than seedlings from PS IAA in both roots and coleoptiles (by 68% and by 25% respectively) (Fig. 8E, F), compared to seedlings from seeds germinating under salt-free conditions. In IAA primed seedlings, CAT activity increased remarkably in maize seedlings exposed to salt stress (300% in roots and 217% in coleoptiles) (Fig. 8E, F), in comparison with salt-stressed seedlings of UPS. For GPX activity, no significant (*p* > 0.05) change was found in roots (Fig. 8G) under all the germination conditions (priming/salt treatment). However, different patterns were recorded for coleoptile GPX activity. Without IAA priming, seedlings grown in saline media displayed the lowest GPX activity (by 61%), compared to 0 mM NaCl (Fig. 8H), whereas, when primed with IAA, GPX activity of coleoptiles was increased by ca. 280% under both saline and non-saline conditions (Fig. 8H).

### Correlation analysis and Principal Component Analysis (PCA)

**Fig. 9:**
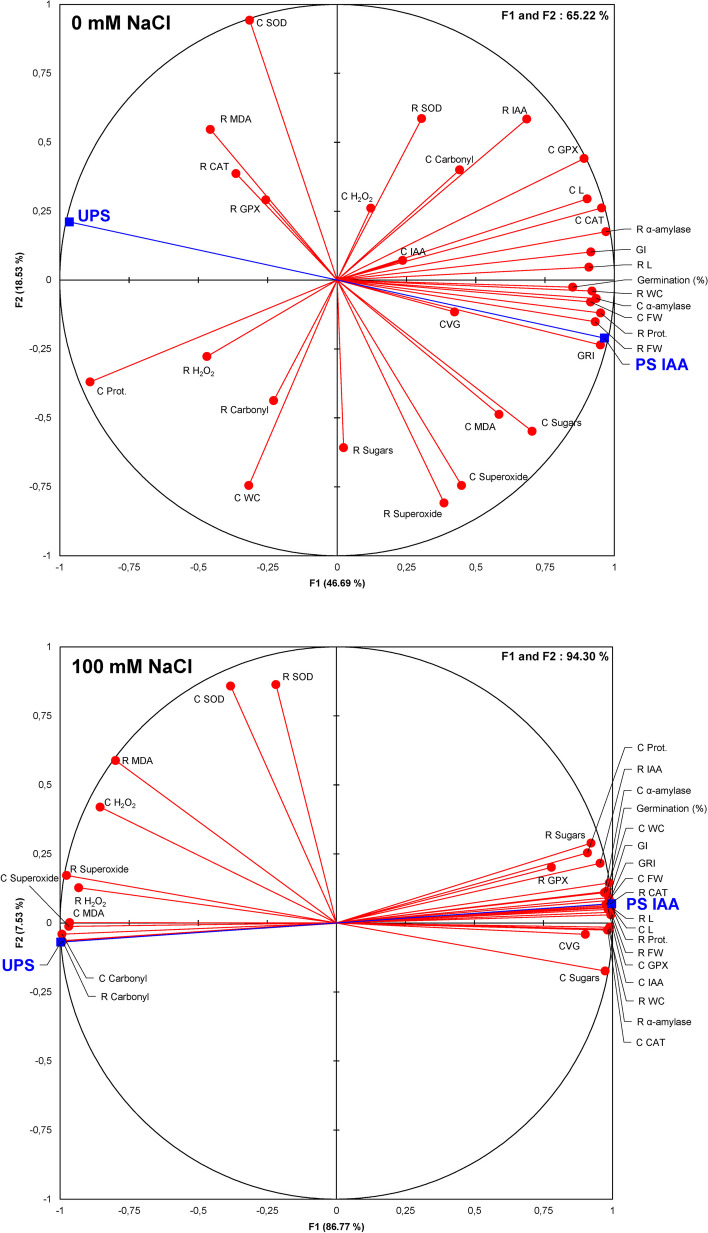
Principal Component Analysis (PCA) for conditions of 0 mM NaCl and 100 mM NaCl. All studied parameters, along with the different seed priming treatments, are represented on the F1-F2 principal factorial plane. This representation explains 93.32% of the total variance for the PCA under 100 mM NaCl and 70.46% under 0 mM NaCl. Abbreviations: UPS: UnPrimed Seeds; PS IAA: Primed Seeds with IAA; GI: germination index; GRI: germination rate index; CVG: coefficient of velocity of germination; R L: root length; C L: coleoptile length; R FW: root fresh weight; C FW: coleoptile fresh weight; R WC: root water content; C WC: coleoptile water content; R Prot.: root protein content; C Prot.: coleoptile protein content; R H_2_O_2_: root hydrogen peroxide content; C H_2_O_2_: coleoptile hydrogen peroxide content; R MDA: root malondialdehyde content; C MDA: coleoptile malondialdehyde content; R Carbonyl: root carbonyl content; C Carbonyl: coleoptile carbonyl content; R SOD: root superoxide dismutase activity; C SOD: coleoptile superoxide dismutase activity; R CAT: root catalase activity; C CAT: coleoptile catalase activity; R GPX: root guaiacol peroxidase activity; C GPX: coleoptile guaiacol peroxidase activity

To further assess the positive effects of IAA priming under both saline and non-saline conditions, correlation analysis (Table 1) and PCA (Fig. 9) were performed. Results showed multiple significant positive correlations, especially under salt stress combined with IAA priming for almost all the parameters we considered (GI, GRI, CVG, root length, coleoptile length, root and coleoptile biomass, root and coleoptile hydration, root sugar content, coleoptile sugar content, root α-amylase activity, coleoptile α-amylase activity, root IAA content, coleoptile IAA content, root protein content, coleoptile protein content, root CAT activity, coleoptile CAT activity, and GPX activities). When examining these correlations, seed priming with IAA combined with salt stress was found to positively correlate to all the aforementioned parameters. This treatment also prevented ROS damage as reflected by the reduced values for root and coleoptile H_2_O_2_ content, root and coleoptile superoxide content, root and coleoptile MDA content, root and coleoptile carbonyl content (Table 1, Fig. 9).

**Table 1:**
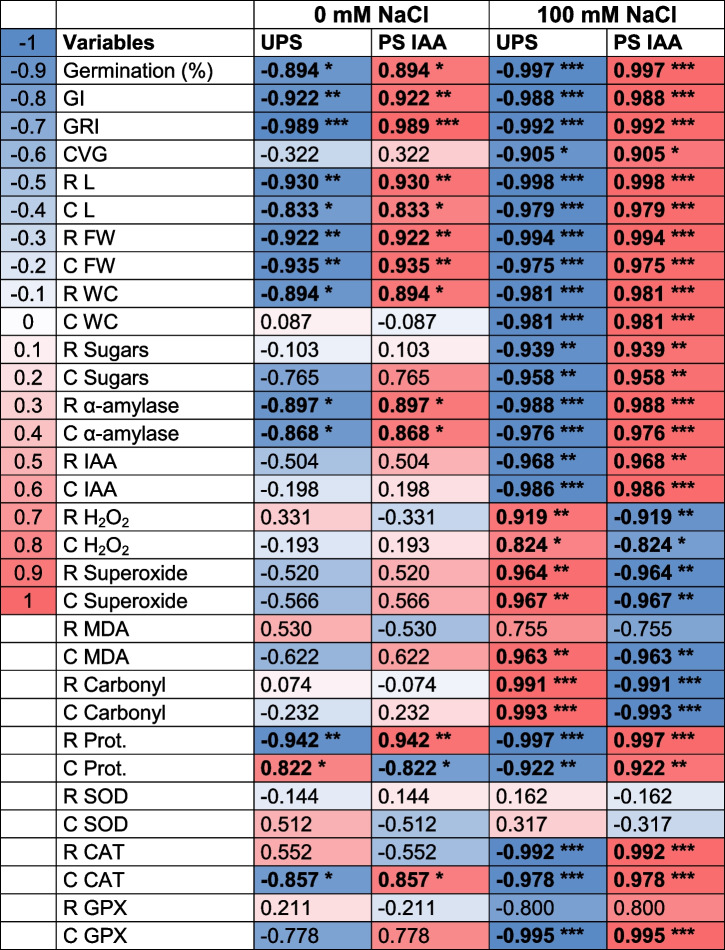
Pearson’s correlation matrix analyzing the correlations between analyzed parameters and the applied treatments in the roots (R) and coleoptiles (C) of maize seedlings. The applied treatments include UnPrimed Seeds (UPS) and Primed Seeds with Indole-3-Acetic Acid (PS IAA), both tested under conditions of 0 mM and 100 mM NaCl. The values presented in the correlation matrix represent correlation coefficients (*r*). Negative correlations are shown in blue, while positive correlations are shown in red. The color intensity is proportional to the correlation coefficient value (see scale on the left of the table). Values in bold with asterisks indicate statistically significant correlations. The significance levels are denoted by *, **, and *, indicating significant correlations at significance levels of *alpha* ≤ 0.05, *alpha* ≤ 0.01, and *alpha* ≤ 0.001, respectively. Germination (%): germination percentage; GI: germination index; GRI: germination rate index; CVG; coefficient of velocity; RL: root length, CL: coleoptile length, RWF: root fresh weight; CWF: coleoptile; RWC: root water content; CWC: coleoptile water content; R sugars: root sugar content, C sugars: coleoptile sugar content, R α-amylase: root α-amylase activity, C α-amylase: coleoptile α-amylase activity, R IAA: root IAA content, C IAA: coleoptile IAA content, R H_2_O_2_: root H_2_O_2_ content, C H_2_O_2_: coleoptile H_2_O_2_ content, R Superoxide: root superoxide content, C Superoxide: coleoptile superoxide content; R MDA: root MDA content, C MDA: coleoptile MDA content, R Carbonyl: root carbonyl content; C Carbonyl: coleoptile carbonyl content; R Prot.: root protein content, C Prot.: coleoptile protein content, R SOD: root SOD activity; C SOD: coleoptile SOD activity; R CAT: root CAT activity, C CAT: coleoptile CAT activity; R GPX: root GPX activity, C GPX: coleoptile GPX activity

It is also worth mentioning that the most interesting correlations were obtained under saline conditions, suggesting that the beneficial effects of seed priming were most pronounced in these circumstances. It is worth noting that these analyses underscored the positive impact of seed priming with IAA, assisting *Z. maize* seedlings in rapidly recovering from salt-induced oxidative stress damages to better survive under salinity.

## Discussion

Achieving seed germination is essential for successful seed emergence and seedling establishment. However, germination is the most sensitive stage in the plant life cycle under salinity, affecting both sensitive species (glycophytes) and salt-tolerant species (halophytes) [14]. Seed priming with phytohormones has proved as a useful agronomic strategy to enhance seed vigor and germination under both stressful and normal conditions [51]. In this study, we postulate that auxin (IAA) seed priming can swiftly regulate the physiological and biochemical mechanisms activated early in maize seedlings during the coleoptile stage under saline conditions. We further propose that post-priming mechanisms, especially those related to ROS-scavenger systems, would confer maize with enhanced and sustained tolerance to salt stress.

### IAA signal priming enhances maize germination

Our data indicate that IAA seed treatment significantly improved overall germination indices in both saline and non-saline conditions compared to unprimed seeds. Under saline conditions, IAA-primed seeds displayed the highest GI, GRI, and CVG, reaching the maximum germination percentage 6 d after seed sowing. This response, together with the observed increase in radicle and coleoptile lengths from IAA-primed seeds, suggests that IAA is an efficient priming factor for enhancing germination performance and seedling emergence, especially under salt stress. These observations suggest that auxin may certainly be involved in the maintain of ion homeostasis under salinity stress by regulating ion fluxes, compartmentalization, and accumulation. So that, the beneficial effects of IAA are enhanced under saline conditions compared to non-stress conditions [52]. In this regard, it has been postulated that priming seeds with IAA mitigates the detrimental effect of salinity in Faba bean seedlings by inhibiting excessive accumulation of Na⁺ in the roots and at the same time, enhancing potassium and calcium nutrition [53].

Many genes expressed during priming, encode for membrane proteins, which probably provided beneficial effects to the maintenance of cell functions and regulation of the ions present in greater amounts during salt stress [54]. Generally, salt stress activates the salt overly sensitive (SOS) pathway which is a key determinant of salt stress tolerance and is highly involved in the regulation of plant ion homeostasis [55]. On the basis of our findings, we propose that IAA, in the primed seed itself, stimulates the expression and activity of SOS1 (Na^+^/H ^+^ antiporters) and other SOS pathway components to alleviate Na^+^ toxicity and improve salt tolerance. Little is known about IAA/SOS interaction in the context of priming. Hence, further investigations are needed to unravel the possible interaction between IAA and SOS component during the priming phenomenon.

Iqbal and Ashraf [26] also documented such a beneficial effect of IAA seed priming on wheat germination, as well as on germination rate, index, and speed in cotton under high salt stress [56]. Mung bean seed germination was also significantly improved by IAA priming under salinity [57], though a negative effect of IAA seed priming was observed in *A. thaliana* [58]. Ashraf et al. [59] reported that the impact of delayed germination caused by salt stress can be reversed by using IAA as priming agent in wheat.

It is well established that IAA responses can vary by species and are largely influenced by IAA concentrations [60]. Considering the interactions between IAA signals and other phytohormones, Zhao et al. [56] assumed that IAA priming might enhance seed germination by modulating endogenous hormone levels, including GA, ABA, and IAA itself. IAA role in improving seed vigor and germination is further highlighted by its regulation of sugar metabolism and starch synthesis. Consequently, IAA is believed to be intricately linked to grain development and nutrient mobilization in seeds, likely correlating with early gene expression that facilitates plant defense mechanisms, even under stress [61]. More recent insights from Xue et al. [62] on maize germination regulatory network suggest that auxin positively influences maize germination by activating cyclin-dependent kinase (CDK) enzymes. These enzymes mediate cell progression during the cell cycle, and auxin has been shown to elevate transcription levels of specific cyclins, including *CycD2;1*, *CycD4;1*, and *CycD5;2*, during the initial hours of germination. In this way, Khan et al. [63] found that auxin modulates seed germination during salt stress by enhancing a membrane-bound transcription factor (NTM2) resulting in NTM2 IAA_30_ gene overexpression greatly involved in plant tolerance to salinity.

### IAA signal priming facilitates early maize seedling establishment

The present study reveals that in the absence of priming, salt treatment sharply impacted seedling growth and hydration, with roots, directly exposed to NaCl, being more affected than shoots. This is likely linked to higher Na^+^ accumulation in the roots. Similar observations were reported in maize where dry weight, water uptake, and both root and shoot lengths experienced considerable reductions, accompanied by significant Na^+^ accumulation, especially in the roots [64]. In contrary, seedlings from IAA-primed seeds exhibited a markedly improved response. There is clear evidence that auxin induces H^+^ excretion, resulting in tissue elongation of soybean hypocotyls [65]. Other studies reported that auxin regulates gene expression in the hypocotyl of soybean seedlings by enhancing RNA levels [65]. This reflects metabolic activation leading to the stimulation of root growth which may probably result in higher germination performance [56]. Our findings confirm that seed priming effectively mitigated the adverse effects of salt treatment by restoring both seedling growth and water uptake. Ulfat et al. [66] also found that IAA priming of wheat seeds markedly enhanced growth and development of wheat plants under drought conditions, leading to an increased grain yield.

Our data support recent studies of Ghanbari and Saeedipour [67] pointing the beneficial effect of IAA seed priming on maize growth and osmotic adjustment under saline conditions. Zhao et al. [56] suggested that IAA likely boosts seedling growth by modulating the sucrose pathway and augmenting photosynthetic activities under salinity. This was further supported by Zhu et al. [68] who investigated the interplay between IAA and photosynthesis components in *Brassica napus*. Yet, the detailed mechanisms through which IAA priming alleviates salt stress remain elusive. One likely explanation might be IAA potential to enhance chlorophyll production and facilitate stomatal movement, which in turn optimizes energy efficiency during photosynthesis, leading to improved plant growth [23].

Seedlings from primed seeds exhibited varied responses under both saline and non-saline conditions. This suggests that maize seeds might perceive IAA treatment differently. It seems that IAA priming is more effective under salt stress, allowing maize seeds to gain higher benefits from IAA treatment when exposed to salinity compared to standard conditions (absence of salt stress). One of the possible mechanisms by which priming improves stress tolerance is the prompt and amplified activation of a plethora of signal events upon the exposure to subsequent stress [69]. Here, we suggest that the positive influence of IAA seed priming on pre-germinative metabolism ensures proper germination and, consequently, early and better seedling establishment. This effect may be associated with the generation of important and specific IAA signaling pathways, probably triggered during priming, retained until later stages of development and amplified under stressful conditions. We, therefore, checked the effect of seed treatment with IAA on the endogenous content of IAA and explored the correlation with sugar accumulation and α-amylase activity in salt-stressed seedlings.

### IAA signal priming initiates a cross-talk between carbohydrates and endogenous IAA

In the present investigation, salt stress caused an early and noticeable reduction in endogenous IAA in maize seedlings derived from unprimed seeds and grown under salinity. Our findings are supported by Pandey et al. [70] who reviewed that auxin accumulation decreased under salt stress which greatly impacted root architecture. Similarly, salt stress may downregulate auxin biosynthesis genes, resulting in reduced auxin levels. For example, Du et al. [71] reported that salt stress decreased auxin biosynthesis by decreasing the expression of six *YUCCA* genes in *Oryza sativa* seedlings. However, presoaking of seeds with IAA effectively alleviated the toxicity of salt stress and so that significant increase of IAA content was recorded in maize seedlings issued from IAA primed seeds and grown under saline conditions. In accord with these findings, Godoy et al. [72] postulated that seed priming with tryptophan, main precursor of the IAA biosynthesis pathways, significantly increased IAA production during wheat germination, which is crucial for seedling establishment during the first days of heterotrophic growth. We also found, in the present investigation, a trend of increasing root and shoot size associated with IAA seed treatment. Thus, seed priming with IAA ensures better performance in germination and subsequent seedling growth, and is potentially effective for seeds germinated in salt stress conditions [72]. At the same time, we found that seedlings from primed seeds with IAA exhibited the highest level of sugar accumulation accompanied by the highest α-amylase activity in both roots and coleoptiles. Thus, one may reasonably suggest that the presence of IAA in seeds (during priming) promoted the metabolic activation crucial to break down the starch reserve. This may be considered as the initial priming signal which occurs and imprinted first in seeds and triggered when subjecting seedlings to subsequent salt stress. This hypothesis is supported by Gutierrez et al. [73] who reported that the starch during the early stages seems to be more abundant as the seed is not yet sufficiently hydrated. However, during the next stages, starch decreases and it is made available to the seedlings in the form of glucose, fructose and sucrose, which not only serve an energy source but also as paramount regulators of auxin gene expression [31]. This disagrees to some extent with the findings of Queiroz et al. [36] reporting that IAA seed priming impacted the activity of starch metabolism enzymes in primed seeds of soybean. However, our findings agree with those of Abdel et al. [53] who found that IAA is an efficient regulator of carbohydrate metabolism in Faba bean seeds.

### IAA signal priming governs early ros detoxification in maize seedlings

Reactive oxygen species (ROS) such as hydrogen peroxide (H_2_O_2_), superoxide radical (O_2_^•−^), hydroxyl radical (^•^OH), and singlet oxygen (^1^O_2_) can be harmful when excessively produced [74]. However, ROS are increasingly viewed as crucial signaling molecules that play regulatory roles in plant growth, development, and stress tolerance enhancements [12]. Several studies highlighted the paramount role of ROS and phytohormone for salt stress tolerance in plants [33, 75]. The interplay between auxin and ROS signaling integrates them as central elements within hormonal signaling networks, predominantly under stress conditions [76]. An intriguing question that emerges is how auxin (IAA) and ROS might interact during the priming process, facilitating maize seedlings to thrive under intense salinity.

In this investigation, maize seedlings originating from unprimed seeds and subjected to salt stress displayed elevated H_2_O_2_ levels in both roots and coleoptile, an effect closely aligned with enhanced lipid peroxidation protein oxidation. IAA-primed seeds germinated under saline conditions exhibited significantly reduced H_2_O_2_, superoxide, carbonyl and MDA concentrations compared to the unprimed state under salinity. It is noteworthy that H_2_O_2_ levels in roots from IAA-primed seeds surpassed those in coleoptiles, suggesting that salt signals are first sensed by roots before being relayed to aerial parts. Our findings are consistent with several other studies [77, 78] stating that H_2_O_2_ is an effective mediator of auxin signaling in plant roots. Moreover, Zhou et al. [79] reported that exogenous H_2_O_2_ treatment resulted in the redistribution of PIN1 and PIN2 Arabidopsis roots leading to auxin gradient arrangement. Furthermore, H_2_O_2_ has been also identified as a mediator of auxin-regulated cell elongation in maize seedlings under salt stress [80]. Another study demonstrated H_2_O_2_ role in stimulating auxin-regulated gravitropism in maize roots [81]. Ivanchenko et al. [82] noted that auxin augmented H_2_O_2_ accumulation in *Solanum lycopersicum* roots, resulting in root cell elongation. These reports imply that despite its toxic nature, H_2_O_2_ might act as central signaling entities during seed germination priming. Prior research has highlighted the role of oxidative stress markers in the priming process [7, 22] which could partially elucidate the enhanced growth observed in roots from IAA-primed seeds. Our data indicate that, in the presence of IAA, maize seedlings may develop a better root system, which likely ameliorates water uptake, thus maintaining osmotic balance between roots and leaves. The present study supports our previous investigations that seed priming with a signal molecule such as H_2_O_2_ [7] and SA [22] easily improved ROS detoxification in plants. Yet, to ascertain this hypothesis, molecular and epigenetic analyses are needed.

The observed reduction in oxidative stress severity in maize coleoptiles might support photosynthesis establishment through IAA signaling, a hypothesis echoed by Zhu et al. [68] in *B. napus* under saline conditions. This suggests a potential metabolic interplay between IAA and photosynthesis. Numerous studies have shown that exogenously applied or priming-induced IAA increases chlorophyll content, photosynthetic rate, sugar accumulation, and stomatal conductance. Collectively, these may serve as ROS scavengers and membrane protectors [61, 83].

### IAA signal priming governs early antioxidant defense in maize seedlings

The capacity of plants to counteract ROS accumulation is indicative of their tolerance to oxidative stress under saline conditions [7]. Antioxidant enzymes, including superoxide dismutase (SOD), catalase (CAT), and guaiacol peroxidase (GPX), play a pivotal role in defending plants against oxidative damage during salt stress [84]. In the current investigation, seedlings from IAA-primed seeds exhibited higher protein levels compared to those from unprimed seeds. Besides, a significant activation of the antioxidant enzymatic systems was observed in both roots and shoots of the IAA-primed seeds. In agreement with Ma et al. [85], our data also showed enhanced antioxidant defense in rice plants from IAA-treated seeds under alkaline conditions. Similar observations were made for IAA-primed *V. faba* plants under intense salinity [53].

We demonstrated differential functions of antioxidant enzymes in roots and shoots. Specifically, CAT was more pivotal for root protection, GPX was crucial for coleoptile defense, while SOD exhibited uniform activity in both. Similarly, Ma et al. [85] found increased CAT, SOD, and POD activities in rice seed roots primed with IAA, with no significant variations observed in leaves. This pattern, indicating a priming effect on antioxidant dynamics, was also identified in salt-stressed barley sourced from silicon-primed seeds [12].

IAA-primed maize seedlings successfully modulated defense strategies to better counteract subsequent salt exposure. Prior research on cauliflower proposed that antioxidant systems might be pre-activated within the seed, facilitating adaptive responses under future salinity challenges [7]. Mir et al. [86] further suggested that IAA in seeds regulates antioxidant enzyme dynamics in *Brassica juncea* under salt stress. Literature highlights that seed priming with signaling molecules, such as H_2_O_2_ [7], ethylene [22], and SA [11], promotes ROS detoxification and bolsters antioxidant defense under saline conditions. Based on this research, we further strengthen the positive correlation between IAA seed priming and the associated IAA signaling pathway, underpinning plant proactive defense against oxidative stress.

**Fig. 10:**
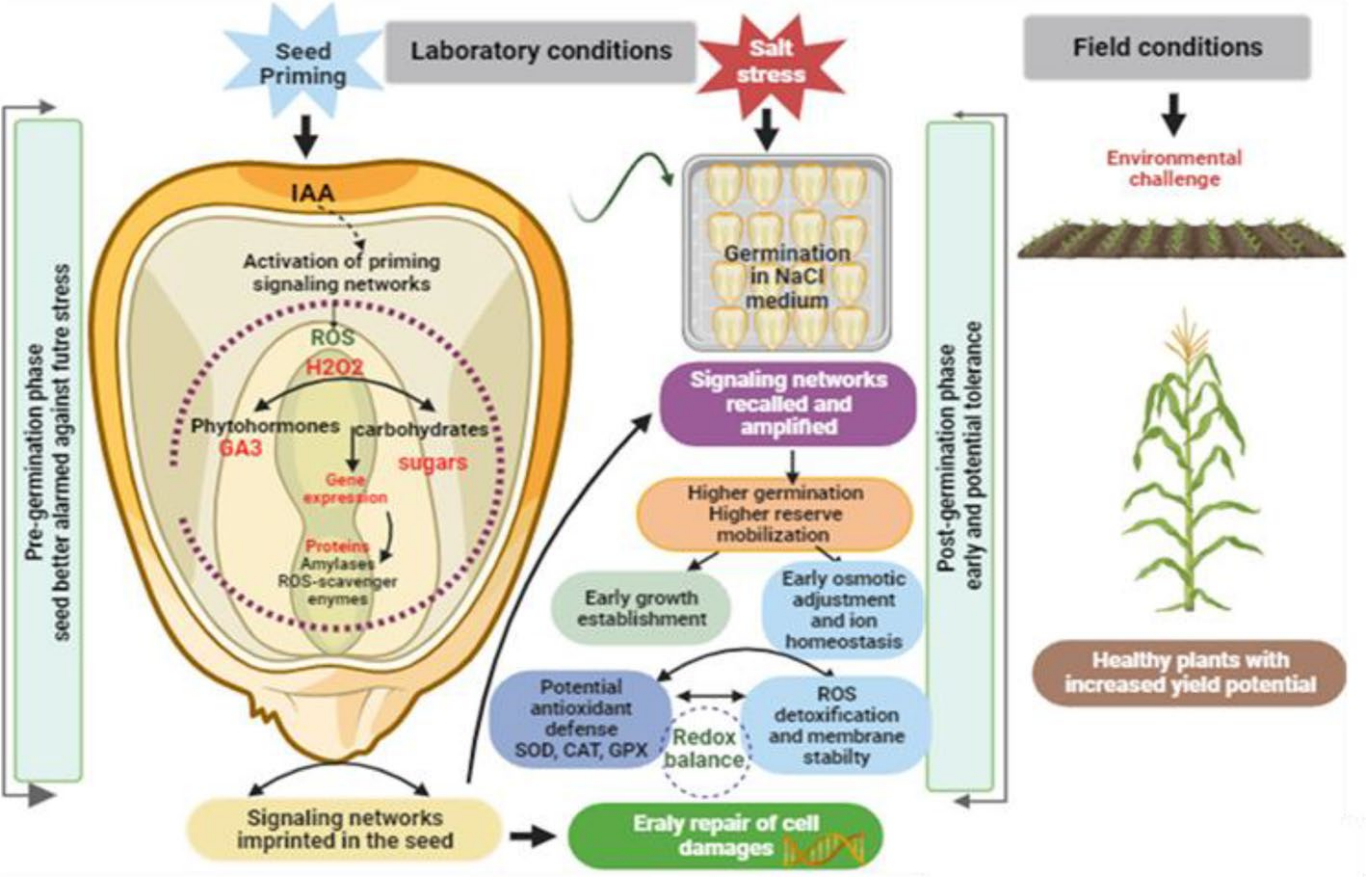
Schematic representation illustrating the potential synergistic interplay mediated by IAA priming in maize seeds in tandem with various signaling networks, especially under salt stress conditions. The diagram is segmented into distinct developmental phases: Seed Priming Phase: Here, the seed undergoes priming with IAA, which activates priming signaling networks within the seed. This priming leads to the modulation of ROS and H_2_O_2_ levels, interaction with phytohormones like GA_3_, and stimulation of certain gene expressions linked with carbohydrates and sugars. Additionally, the seed’s protein production, particularly ROS-scavenger enzymes, is enhanced, leaving an imprint of these signaling networks. Laboratory Conditions: Under controlled environments, the seed experiences salt stress. The priming ensures the seed’s germination in the NaCl medium. The pre-established signaling networks are recalled and intensified, promoting higher germination rates and better mobilization of stored reserves. This phase also witnesses the early establishment of growth patterns, adjustments for osmotic stress, ion homeostasis, and a heightened potential for antioxidant defense mechanisms like SOD, CAT, and GPX. These adaptations foster a balance in the redox states and fortify membrane stability, aiding in the rapid repair of cell damages. Field Conditions: Upon transitioning to field conditions, the seedling would likely face various environmental challenges. However, with the speculated influence of IAA priming, it is hypothesized that the plant would exhibit resilience, potentially demonstrating robust early growth. This would aid in effectively counteracting early developmental challenges, and, given the ideal circumstances, could lead the plant to mature into a healthy specimen with a promising yield potential. The diagram offers a comprehensive overview of how IAA priming potentially interlinks with multiple signaling pathways, paving the way for a maize seedling’s fortified response to salt stress from its germination stage right up to its growth in challenging field conditions, resulting in enhanced yield potential

In summary, the results of this study indicate that IAA priming enhances maize resilience to salinity at early developmental stages by orchestrating a symphony of interactions between IAA and various signaling cascades. We postulate that IAA in seeds first engages with germination regulators, like GA_3_ and carbohydrates. Then, IAA assimilated by seeds during priming may augment osmolyte production, vital for ion balance. Lastly, the interplay between ROS and IAA is crucial for antioxidant defense (Fig. 10). We hypothesize that these mechanisms serve as indicators of the ‘priming memory’ conferred by IAA, conferring to maize seedlings better salt stress tolerance. How auxin interacts with multiple signaling pathways remain enigmatic. Hence, further research will allow to better understand the nuances of IAA priming in the quest for sustainable agriculture.

## Conclusion

This study provides compelling evidence of the transformative role of IAA in seed priming, underscoring its capacity to modulate intricate interactions among numerous signaling pathways right from the seed phase. Such interactions, as revealed in our investigation, imbue maize seedlings with an augmented resilience, equipping them to effectively confront and endure subsequent encounters with salt stress. The insights gleaned from this research not only shed light on the intricate biochemical interplays at work but also open up promising avenues for leveraging IAA potential in sustainable agricultural practices, especially in salt-affected areas. As the global agricultural community pivots towards greener technologies aiming to optimize yields, our findings underscore the significance of harnessing natural compounds like IAA to pave the way for innovative, sustainable solutions in the face of environmental challenges.

## Data Availability

All relevant data can be found in the manuscript.
